# UNDERSTANDING EVERYDAY NEEDS OF OLDER ADULTS WITH MACULAR DEGENERATION OR GLAUCOMA

**DOI:** 10.1093/geroni/igad104.0127

**Published:** 2023-12-21

**Authors:** Kara Mumma, Elena Remillard, Tooba Umar, Divya Bhookya, Wendy Rogers

**Affiliations:** Georgia Institute of Technology, Atlanta, Georgia, United States; Georgia Institute of Technology, Atlanta, Georgia, United States; College of Applied Health Sciences, University of Illinois Urbana-Champaign, Champaign, Illinois, United States; College of Applied Health Sciences, University of Illinois Urbana-Champaign, Champaign, Illinois, United States; University of Illinois Urbana-Champaign, Champaign, Illinois, United States

## Abstract

Older adults with long-term vision loss may experience challenges in their everyday activities. Different types of vision loss may cause different problems. For example, macular degeneration leads to a loss of foveal vision whereas glaucoma leads to peripheral vision loss. As part of the Aging Concerns, Challenges, and Everyday Solution Strategies (ACCESS) study, we are using a mixed-methods approach to explore the unmet needs of older adults with these two conditions to provide guidance for interventions. We will present insights from 51 older adults (mean age = 69, SD = 5.5) who have had serious difficulty seeing for an average of 42 years (SD = 24.3) due to either glaucoma (n = 28), macular degeneration (n= 19), or both conditions (n = 4). We will present participants’ difficulty ratings for a wide range of everyday activities as well as initial themes and illustrative quotes from interview data on challenges and response strategies for their most difficult activities (e.g., shopping, traveling for leisure, dressing). For example, it can be difficult to navigate through a crowded store and read details on labels when shopping. Additionally, flying was described as frustrating and stressful because airports can be unfamiliar and require arrangements (e.g., transportation to/from, guidance to the gate). Moreover, when getting dressed, participants said that they rely on touch to differentiate between clothes; however, color coordination and matching can be very difficult. Data provide insights on unmet needs among people aging with vision loss and illuminate opportunities for technology innovation.

